# Utility of oropharyngeal real-time PCR for *S. pneumoniae* and *H. influenzae* for diagnosis of pneumonia in adults

**DOI:** 10.1007/s10096-016-2829-z

**Published:** 2016-11-07

**Authors:** A. Bjarnason, M. Lindh, J. Westin, L.-M. Andersson, O. Baldursson, K. G. Kristinsson, M. Gottfredsson

**Affiliations:** 1 Faculty of Medicine, University of Iceland, Reykjavik, Vatnsmyrarvegi 16, 101 Reykjavik, Iceland; 2 Departments of Medicine, Microbiology and Virology, Landspitali University Hospital, 101 Reykjavik, Iceland; 3 Department of Infectious Diseases/Clinical Virology, University of Gothenburg, Medicinaregatan 3a-5b, 40530 Gothenburg, Sweden; 4 Division of Infectious Diseases, Landspitali University Hospital, Fossvogur, 108 Reykjavik, Iceland

## Abstract

A lack of sensitive tests and difficulties obtaining representative samples contribute to the challenge in identifying etiology in pneumonia. Upper respiratory tract swabs can be easily collected and analyzed with real-time PCR (rtPCR). Common pathogens such as *S. pneumoniae* and *H. influenzae* can both colonize and infect the respiratory tract, complicating the interpretation of positive results. Oropharyngeal swabs were collected (*n* = 239) prospectively from adults admitted to hospital with pneumonia. Analysis with rtPCR targeting *S. pneumoniae* and *H. influenzae* was performed and results compared with sputum cultures, blood cultures, and urine antigen testing for *S. pneumoniae*. Different Ct cutoff values were applied to positive tests to discern colonization from infection. Comparing rtPCR with conventional testing for *S. pneumoniae* in patients with all tests available (*n* = 57) resulted in: sensitivity 87 %, specificity 79 %, PPV 59 % and NPV 94 %, and for *H. influenzae* (*n* = 67): sensitivity 75 %, specificity 80 %, PPV 45 % and NPV 94 %. When patients with prior antimicrobial exposure were excluded sensitivity improved: 92 % for *S. pneumoniae* and 80 % for *H. influenzae*. Receiver operating characteristic curve analysis demonstrated for *S. pneumoniae*: AUC = 0.65 (95 % CI 0.51–0.80) and for *H. influenzae*: AUC = 0.86 (95 % CI 0.72–1.00). Analysis of oropharyngeal swabs using rtPCR proved both reasonably sensitive and specific for diagnosing pneumonia caused by *S. pneumoniae* and *H. influenzae*. This method may be a useful diagnostic adjunct to other methods and of special value in patients unable to provide representative lower airway samples.

## Introduction

The microbial etiology of pneumonia often remains undetected despite extensive diagnostic testing [1]. Blood cultures lack sensitivity and obtaining representative lower respiratory tract samples can be challenging [2]. An etiologic diagnosis allows targeted antimicrobial treatment, a matter of increasing importance as resistance rates increase [3].

*Streptococcus pneumoniae* (SP) and *Haemophilus influenzae* (HI) are common causes of pneumonia but also colonize the upper respiratory tract at rates ranging from 1 to 76 %, highest in young children [4–6]. Lower respiratory tract samples such as sputum are thus preferred for diagnostic purposes.

Multiplex real-time PCR (rtPCR) panels have become established in the diagnosis of respiratory tract infections but primarily used to identify viruses and “atypical” bacteria [7]. Due to easily collected samples and the high sensitivity of PCR-based methods, upper airway sampling is often used even when a lower respiratory tract infection is suspected. The likelihood of a causal association is high if an organism not known to colonize the respiratory tract is detected in the setting of pneumonia.

Sputum and nasopharyngeal samples have been examined with PCR targeting SP and HI applying quantitative thresholds to discern infection from carriage [8–10]. However, analyzing sputum does not avoid the problems associated with obtaining high quality samples [9, 11]. Despite closer anatomical proximity to the site of infection, and study results suggesting lower carriage rates in this area, studies examining oropharyngeal sampling in pneumonia are lacking [12, 13].

The authors are not aware of any previous studies examining the utility of rtPCR examining HI from the oropharynx or Spn9802 as a target for SP in the setting of pneumonia. Different PCR targets for SP have been proposed but problems with specificity can occur [13, 14].

The aim of this study was to examine the utility of rtPCR from oropharyngeal swabs for the etiologic diagnosis of pneumonia caused by SP and HI in adults by comparing rtPCR results with other, established etiological tests and applying different cycle threshold (Ct) cut-off values to quantitatively differentiate carriage from infection.

## Materials and methods

### Patient inclusion and etiological testing

Data was derived from patients admitted with pneumonia from December 2008 to November 2009 to Landspitali University Hospital, Reykjavik, Iceland [15]. All participants had a new chest X-ray infiltrate and clinical symptoms of pneumonia [16]. Patients with hospital-acquired pneumonia were excluded. Cultures were collected prior to antibiotic administration in hospital. Only high quality sputum was included [17]. Blood cultures were collected, incubated and cultured using standard methods at our center and susceptibility testing performed using the Clinical and Laboratory Standards Institute methods and criteria [18]. Urine antigen testing (UAT) for SP was performed using a commercially available kit (Binax NOW Streptococcus pneumoniae). An oropharyngeal swab sample (sterile rayon tipped swabs, COPAN Italia) was collected for rtPCR. Pneumonia Severity Index (PSI) and CURB-65 scores were calculated [19, 20]. Participant reported antimicrobial use during the 14 days prior to admission was recorded.

### Real-time PCR

Nucleic acid from 200 μL specimens was extracted with QIAmp DNA Blood Mini Kit (QIAGEN) and the MagNa Pure Compact Nucleic Acid Isolation Kit I (Roche Diagnostics). The nucleic acids were eluted in 100 μL volume, and 5 μL used for rtPCR. RtPCR was performed with an ABI 7900 384-well system (Applied Biosystems, Foster City, CA, USA) in eight parallel 20 μL reactions containing Universal Mastermix (ABI), including oligonucleotides targeting the omp6 gene of HI and the Spn9802 fragment of SP. Abdeldaim et al. utilized the same targets [21, 22], but in order to obtain shorter amplicons and greater specificity new primers and probes were designed. For both bacteria primers were altered to decrease amplicon length compared with the referenced method to achieve a more effective PCR. For HI two mismatches for *Haemophilus haemolyticus* were introduced in the probe to increase specificity. The specificity of rtPCR for SP and HI was tested using reference samples containing *Streptococcus mitis, Streptococcus oralis* and *Streptococcus sanguinis*, and *H. haemolyticus* and *Haemophilus parainfluenzae* respectively. No cross reaction was noted with either comparison.

Thus, the omp6 gene of HI was amplified by forward primer CTAACAACGATGCTGCAGGCA, reverse primer GTGTTATAACGTTGTTGAAGATCAGC and probe, NED-ATGGTGCTGCTCAAA-MGB (MGB, minor groove binder); and the Spn9802 fragment of SP with forward primer TTTCTGGATAGAGGGAGTATCCGA, reverse primer TTACCAACCTACTCATCTTCTCACCA, and probe FAM-CAAAGTTAATACCGCCCTC-MGB. After a reverse transcription step at 46 °C for 30 min followed by 10 min of denaturation at 95 °C, 45 cycles of two-step PCR was performed (15 s at 95 °C, 60 s at 58 °C). A pUC57 plasmid carrying target sequences was used in each run parallel with patient specimens to verify the performance of PCR (master mix control). In addition, one positive control was processed with each set of samples, from extraction of nucleic acids through the detection of amplified products.

### Comparison of different tests

Sensitivity, specificity, positive predictive value (PPV) and negative predictive value (NPV) of rtPCR for SP and HI were calculated using combined and separate results from sputum (SP and HI) and blood cultures (SP only), and urine antigen analysis (SP only) as a reference “gold standard”. For each comparison, only participants with both rtPCR results and the reference test in question available were included. Four different threshold cycle (Ct) cutoffs were applied. For these calculations we assumed that a positive culture or urinary antigen test indicated a definite etiologic diagnosis while cases with positive rtPCR but negative comparative tests indicated colonization.

### Statistical analysis

Data for SP and HI were summarized using receiver operating characteristic (ROC) curves to determine diagnostic efficacy of the rtPCR for different Ct values. Positive rtPCR results were examined and true positives determined using culture or UAT. AUC with 95 % confidence intervals (CI) were determined and optimal Ct cut off determined.

Categorical data was compared using chi-square or Fisher’s exact test as appropriate. Statistical significance was set at two-tailed *P* = 0.05. Continuous data was compared using 95 % CI. Calculations were performed using IBM SPSS Statistics Version 22.0.0.0.

The Landspitali University Hospital ethics committee approved this study which is in accord with the revised Helsinki Declaration. All patients or proxy provided written informed consent.

## Results

The study cohort is described in Table 1. In all 373 pneumonia cases were included but complete sample sets were not universally available. Differences in demographics, disease severity and outcomes between groups providing different samples were analyzed. Availability of PCR swabs was associated with a lower likelihood of ICU care (5.4 vs. 14.2 %, *p* < 0.01) and lower 30-day mortality (2.1 vs. 9.0 %, *p* < 0.01) (Table 1).

**Table 1:** Comparison of underlying characteristics, severity scores and selected blood tests on arrival and outcomes for patients by tests submitted

Characteristic	All patients	PCR swab available	Sputum available	Blood culture available	UAT available	*S. pneumoniae* PCR positive	*H. influenzae* PCR positive
No. (%)	373 (100)	239 (64)	116 (31)	280 (75)	273 (73)	61 (26)^a^	51 (21)^a^
Age, mean (95 % CI)	63.6 (61.6–65.6)	63.8 (61.2–66.4)	62.5 (58.8–66.2)	62.5 (60.1–64.8)	63.4 (61.1–65.7)	65.9 (60.3–69.4)	63.5 (57.6–69.4)
No. (%) male	180 (48)	112 (47)	59 (50)	140 (50)	133 (49)	33 (54)	21 (41)
No. (%) with antibiotics prior to admission	123 (33)	75 (31)	34 (29)	88 (31)	95 (35)	14 (23)	19 (37)
PSI points, mean (95 % CI)	79.9 (76.4–83.5)	79.3 (75.0–83.7)	77.8 (71.6–84.1)	79.9 (75.7–84.1)	78.8 (74.8–82.9)	80.0 (71.9–88.0)	82.1 (72.5–91.7)
CURB-65 points, mean (95 % CI)	1.3 (1.2–1.4)	1.4 (1.2–1.5)	1.3 (1.1–1.5)	1.3 (1.2–1.4)	1.3 (1.2–1.4)	1.4 (1.1–1.7)	1.4 (1.1–1.7)
APACHE II score, mean (95 % CI)	9.9 (9.4–10.5)	9.9 (9.2–10.5)	9.2 (8.3–10.2)	10.3 (9.6–10.9)	9.9 (9.3–10.6)	9.8 (8.5–11.2)	9.9 (8.5–11.3)
CRP, mean (95 % CI)	128 (118–138)	130 (117–143)	128 (110–146)	136 (124–147)	132 (121–135)	135 (109–160)	144 (112–176)
White count, mean (95 % CI)	12.3 (11.7–13.0)	12.5 (12.7–13.3)	13.0 (11.8–14.1)	12.3 (11.5–13.0)	12.3 (11.6–13.1)	14.2 (12.7–15.7)	12.6 (10.8–14.5)
Length of stay, mean (95 % CI) (days)	7.7 (7.2–8.2)	7.8 (7.2–8.4)	7.3 (6.5–8.1)	7.9 (7.3–8.5)	7.9 (7.3–8.4)	6.8 (5.7–7.8)	8.2 (6.5–9.8)
No. (%) who received ICU care	32 (9)	13 (5)	5 (4)	29 (10)	25 (9)	4 (7)	3 (6)
30 Day all cause mortality, No. (%)	17 (5)	5 (2)	3 (3)	13 (5)	12 (4)	1 (2)	1 (2)

*UAT* urinary antigen test for S. pneumoniae, *PSI* pneumonia severity index, *CI* confidence interval, *CURB-65* confusion, urea, respiratory rate, blood pressure, *PCR* polymerase chain reaction

^a^Percentage calculated from those with PCR swabs available

### Rates of SP and HI identification

SP was identified with conventional methods in 30 (12.6 %) and HI in 17 (7.1 %) of 239 patients with rtPCR results available, compared with 22 (16.4 %) and 4 (3.0 %) of 134 patients without rtPCR results, differences that were not statistically significant. Using rtPCR, SP was identified in 61 (25.5 %) cases and HI in 50 (20.9 %). Rates of positive rtPCR were similar in patients with sputum culture, blood culture or UAT results available (Table 2).

**Table 2:** Comparison of detection rates for *S. pneumoniae* and *H. influenzae* with real-time PCR and conventional testing^a^ among patients with both tests available

Measure	No. with test available:		
	Sputum culture	Blood culture	Urinary antigen test^b^
	(*n* = 83)	(*n* = 187)	(*n* = 168)
*S. pneumoniae* identified with:			
Conventional method, no. (%)	17 (20)	5 (3)	21 (13)
Real-time PCR^c^, no. (%)	29 (35)	45 (24)	50 (29)
*H. influenzae* identified with:			
Conventional method, no. (%)	12 (16)	0 (0)	
Real-time PCR^c^, no. %	24 (29)	40 (21)	

*UAT* urinary antigen test

^a^Patients with both real time PCR result and relevant alternative test examined for each type of test. Patients could be included in more than one group when multiple cases were available

^b^UAT is not available for detection of *H. influenzae*

^c^Real-time PCR considered positive at Ct of 45 or below

### Sensitivity and specificity of real-time PCR

For SP overall sensitivity was 87 %, specificity 79 %, PPV 59 % and NPV 94 % amongst patients with all tests available. Results were similar when UAT results were excluded from the analysis. For HI sensitivity was 75 %, specificity 80 %, PPV 45 % and NPV 94 %. Excluding patients with recent antibiotic use increased the sensitivity of rtPCR for both SP and HI (Table 3).

**Table 3:** Sensitivity, specificity, positive predictive value and negative predictive value of PCR identification of *S. pneumoniae* and *H. influenzae* from upper airway swabs positive at varying Ct value cutoffs compared with different reference gold standards

Ct^a^	*S. pneumoniae*				*H. influenzae*			
	Sensitivity	Specificity	PPV	NPV	Sensitivity	Specificity	PPV	NPV
Sputum culture (*n* = 83)								
All	82 %	77 %	48 %	94 %	79 %	81 %	46 %	95 %
40	82 %	79 %	50 %	95 %	79 %	93 %	69 %	96 %
35	59 %	89 %	59 %	89 %	57 %	100 %	100 %	92 %
30	41 %	97 %	78 %	86 %	21 %	100 %	100 %	86 %
Blood culture (*n* = 187)								
All	80 %	77 %	9 %	99 %	–*	79 %	0 %	100 %
40	80 %	79 %	10 %	99 %	–	88 %	0 %	100 %
35	40 %	88 %	8 %	98 %	–	95 %	0 %	100 %
30	40 %	95 %	18 %	98 %	–	99 %	0 %	100 %
Urinary antigen test^b^ (*n* = 168)								
All	67 %	77 %	28 %	95 %	–	–	–	–
40	67 %	79 %	30 %	95 %	–	–	–	–
35	48 %	87 %	33 %	93 %	–	–	–	–
30	24 %	96 %	42 %	90 %	–	–	–	–
SC, BC and UAT, any available^c^ (SP *n* = 228; HI *n* = 214)								
All	70 %	81 %	36 %	95 %	79 %	82 %	23 %	98 %
40	70 %	83 %	38 %	95 %	79 %	92 %	39 %	98 %
35	47 %	91 %	44 %	92 %	57 %	98 %	62 %	97 %
30	27 %	97 %	62 %	90 %	21 %	99 %	75 %	95 %
SC, BC and UAT, all available^c^ (SP *n* = 57 & HI *n* = 67)								
All	87 %	79 %	59 %	94 %	75 %	80 %	45 %	94 %
40	87 %	81 %	62 %	94 %	75 %	91 %	64 %	94 %
35	53 %	86 %	57 %	84 %	25 %	100 %	100 %	86 %
30	40 %	95 %	75 %	82 %	17 %	100 %	100 %	85 %
SC, BC and UAT, all available, patients with prior antibiotics excluded^c^ (SP *n* = 37 & HI *n* = 47)								
All	92 %	80 %	69 %	95 %	80 %	84 %	57 %	94 %
40	92 %	84 %	73 %	95 %	80 %	92 %	73 %	94 %
35	58 %	92 %	78 %	82 %	60 %	100 %	100 %	90 %
30	42 %	96 %	83 %	77 %	20 %	100 %	100 %	82 %

*Ct* threshold cycle, *PPV* positive predictive value, *NPV* negative predictive value, *SC* sputum culture, *BC* blood culture, *UAT* urinary antigen test

^a^Ct is a semi quantitative measurement which relates inversely to the samples’ initial DNA concentration

^b^No patients were positive for *H. influenzae* in blood culture

^c^UAT not available for *H. influenzae*, thus not included in right columns

When single tests were compared with rtPCR, sensitivity and specificity for SP were similar for sputum and blood culture while sensitivity was decreased when compared with UAT. A similar comparison was not possible for HI due to lack of positive blood cultures (Table 3).

**Fig. 1:**
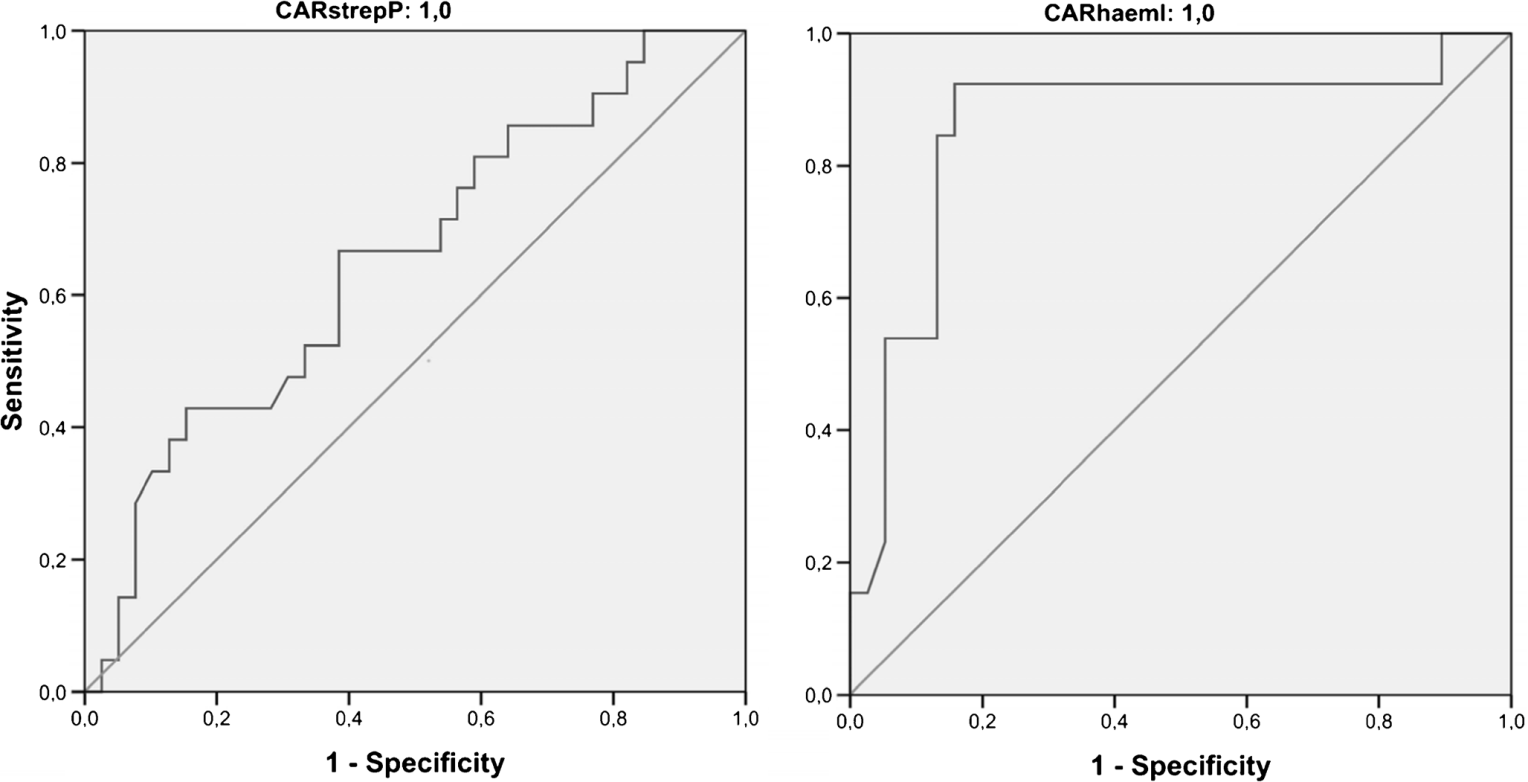
Receiver operating characteristic (ROC) curves comparing Ct results from real-time PCR to results from conventional microbiological testing for *S. pneumoniae* (*left*) (AUC = 0.653; 95 % CI 0.508–0.799) and *H. influenzae* (*right*) (AUC = 0.859; 95 % CI 0.722–0.997)

The ROC curves for SP and HI are shown in Fig. 1. The AUC for SP was 0.65 (95 % CI 0.51–0.80) with an estimated optimal cutoff Ct value of 35 cycles. ROC analysis for HI resulted in an AUC of 0.86 (95 % CI 0.72–1.00) with an estimated optimal Ct cutoff of 33.

## Discussion

This study is the first to examine oropharyngeal rtPCR for HI and target SP with Spn9802 to diagnose pneumonia. Samples from a prospective cohort of consecutive patients requiring hospital admission for pneumonia with a high rate of patient inclusion (95 %) were examined. SP and HI were detected using conventional methods in 14 and 6 % of patients, similar to previous reports [1, 13]. Sensitivity of tests in increasing order were blood culture, UAT (SP only), and sputum culture (Table 2) [23]. The sensitivity of sputum culture is offset by decreased specificity due to risk of contamination from upper airway colonization.

### Performance of real-time PCR

In cases with complete test panels, sensitivity and specificity of rtPCR for SP was 87 and 79 % (Table 3). In comparison, a recent meta-analysis estimated the sensitivity and specificity of UAT for SP to be 69 and 84 % [24]. Allowing incomplete test panels decreased sensitivity, possibly due to false negative results. For HI the sensitivity and specificity were 75 and 80 %. This comparison uses sputum culture for reference as HI was not isolated from blood in any of the included samples.

The PPV for SP was 59 % and NPV 94 % for complete sample sets, and the results for HI were 45 and 94 %. PPV was improved for both pathogens by excluding rtPCR positive at high Ct values, representing less DNA in the initial sample (Table 3). These results may be compared with 91–95 % PPV and 83–91 % NPV for UAT described previously [25, 26].

RtPCR provides quantitative information in the form of Ct values, which may assist in discerning colonization from infection [27]. ROC analysis indicated good accuracy for HI (AUC = 0.86) but relatively poor for SP (AUC = 0.65). Results for rtPCR were improved among patients without prior antibiotic use (Table 3) indicating use of antimicrobials prior to admission may have negatively affected the ROC result.

### Potential impact of cross-reactivity and carriage on specificity

Cross-reactivity with other species may decrease PCR specificity. The pneumolysin gene (*ply*) target for SP may react with alpha-hemolytic streptococci [9, 28]. Targeting the autolysin gene (*lytA*) or Spn9082 may offer higher specificity but *lytA* is also present in other streptococci from the oral flora while the Spn9802 PCR may react to *Streptococcus pseudopneumoniae* [28]. The clinical significance and possible occurrence of *S. pseudopneumoniae* carriage is unclear. Targeting the Spn9802 region may lead to more specific results but has not been utilized in a clinical study of pneumonia etiology to the authors’ knowledge.

The P6 gene has been shown to be a sensitive and specific PCR target for identifying HI [21]. While possible cross-reactivity with related species has been demonstrated, the clinical significance is unclear, a problem compounded by difficulties in discriminating some of these species using conventional methods [29].

As this study did not include a control group background carriage in the population is difficult to assess. Two studies performed in a Nordic setting examined nasopharyngeal cultures from adults with non-infectious conditions at healthcare centers and hospitals and found low carriage rates, i.e. 1–3 % for both SP and HI [5, 8]. Carriage rates increased to 3 and 2 % with PCR analysis [8]. A more recent study examining oropharyngeal samples from community dwelling seniors detected SP in 5 % with culture but 18 % with PCR [30]. However, this study targeted *ply* which may have led to false positive test results due to cross-reactivity with alpha-hemolytic streptococci in the oropharynx [28].

### Comparison with previous studies

In addition to alternative PCR targets, differences in setting and study populations hamper comparison with older studies. Yang et al. examined sputum samples in pneumonia and determined a sensitivity of 90 %, specificity of 80 % and an AUC of 0.87 for identifying SP [9]. It is perhaps not surprising that examining lower respiratory tract samples in a selected cohort of patients would result in a higher AUC. The selected PCR target (*ply*) may also partially explain the higher sensitivity and lower specificity of these results compared with the present study [14].

Strålin et al. compared PCR for SP (*lytA*) and HI (16sRNA with P6 verification) of sputum and nasopharyngeal samples with a composite of reference tests in pneumonia patients. Among patients without prior antibiotic use they found that sputum PCR had a sensitivity of 92 % and specificity of 42 % while nasopharyngeal swabs had a sensitivity of 61 % and specificity of 87 % for SP while the sensitivity for HI was 78 and 80 % [8]. Aside from a slightly higher sensitivity for SP from sputum it is of interest that utilizing sputum and nasopharyngeal samples were not necessarily superior to oropharyngeal swabs for diagnosing pneumonia.

Albrich et al. applied quantitative rtPCR (*lytA)* to examine nasopharyngeal swabs in adult HIV positive pneumonia patients with good results. The sensitivity was 82 % and specificity was 92 % for discerning infection from colonization and the AUC 0.78 for identifying SP. These results are similar to the present study but are difficult to compare due to differences in setting and population [31].

Abdeldaim et al. applied PCR targeting omP6 (HI) to nasopharyngeal aspirates from 166 pneumonia patients. Sensitivity was 97.5 %, specificity 84.1 % and AUC 0.974 when PCR was compared with culture. As these results were derived by comparing analyses on same samples they are difficult to compare with the present results. Of interest in this study confirmatory fucK PCR was performed to establish the specificity of the method [21].

### Lack of a true gold standard

A weakness of this study and other comparable studies is the relatively low sensitivity of the reference methods used for comparison. This decreases sensitivity and specificity and in turn affects AUC [32]. In addition, antibiotic exposure may increase discrepancy between PCR and culture-based results. The improved performance of rtPCR among patients without recent antibiotic use supports this possibility.

### Value of negative results

For both pathogens specificity and NPV were excellent. The application of rtPCR is not constrained to ascertaining etiology but may also be useful for decreasing the clinical likelihood of disease due to certain pathogens. Negative results may also have increased validity in the setting of multiplex-PCR when a clinically reasonable alternative pathogen has been identified. It is doubtful such an interpretation can be made in severely ill patients but might assist in avoiding antimicrobials in certain situations, such as in patients with less severe disease and a high probability of viral infection.

## Conclusions

This study compares rtPCR analysis of oropharyngeal samples with conventional testing for diagnosis of bacterial pneumonia caused by SP or HI. Due to the high inclusion rate the results of this study may be more applicable to other settings than most studies of this nature. The results are comparable to previous studies analyzing high quality sputum. Utilizing different Ct values to quantify positive rtPCR results can assist in discerning colonization from infection caused by SP and HI in the setting of pneumonia. Further studies are required to test these findings and identify patient groups most likely to benefit from these tests.
